# Algorithms for Solving the Equilibrium Composition Model of Arc Plasma

**DOI:** 10.3390/e27010024

**Published:** 2024-12-31

**Authors:** Zhongyuan Chi, Yuzhang Ji, Ningning Liu, Tianchi Jiang, Xin Liu, Weijun Zhang

**Affiliations:** 1 School of Metallurgy, Northeastern University, Shenyang 110819, China; 2210680@stu.neu.edu.cn (Y.J.); jtcedu@163.com (T.J.); 1810574@stu.neu.edu.cn (X.L.); zhangwj@smm.neu.edu.cn (W.Z.); 2 Longhua Technology Group (Luoyang) Co., Ltd., Luoyang 471132, China; lnlh2011@163.com

**Keywords:** arc plasma, equilibrium composition model, HLMA, PV–LMA

## Abstract

In the present study, the Homotopy Levenberg−Marquardt Algorithm (HLMA) and the Parameter Variation Levenberg–Marquardt Algorithm (PV–LMA), both developed in the context of high-temperature composition, are proposed to address the equilibrium composition model of plasma under the condition of local thermodynamic and chemical equilibrium. This model is essentially a nonlinear system of weakly singular Jacobian matrices. The model was formulated on the basis of the Saha and Guldberg–Waage equations, integrated with Dalton’s law of partial pressures, stoichiometric equilibrium, and the law of conservation of charge, resulting in a nonlinear system of equations with a weakly singular Jacobian matrix. This weak singularity primarily arises due to significant discrepancies in the coefficients between the Saha equation and the Guldberg–Waage equation, attributed to differing chemical reaction energies. By contrast, the coefficients in the equations derived from the other three principles within the equilibrium composition model are predominantly single−digit constants, further contributing to the system’s weak singularity. The key to finding the numerical solution to nonlinear equations is to set reasonable initial values for the iterative solution process. Subsequently, the principle and process of the HLMA and PV–LMA algorithms are analyzed, alongside an analysis of the unique characteristics of plasma equilibrium composition at high temperatures. Finally, a solving method for an arc plasma equilibrium composition model based on high temperature composition is obtained. The results show that both HLMA and PV–LMA can solve the plasma equilibrium composition model. The fundamental principle underlying the homotopy calculation of the (*n*−*1*) −th iteration, which provides a reliable initial value for the *n*−th LM iteration, is particularly well suited for the solution of nonlinear equations. A comparison of the computational efficiency of HLMA and PV–LMA reveals that the latter exhibits superior performance. Both HLMA and PV–LMA demonstrate high computational accuracy, as evidenced by the fact that the variance of the system of equations ||***F***|| < 1 × 10^−15^. This finding serves to substantiate the accuracy and feasibility of the method proposed in this paper.

## 1. Introduction

The plasma equilibrium composition refers to the number density of each particle species within the plasma under conditions of local thermodynamic equilibrium and chemical equilibrium. This composition serves as a fundamental condition for determining the physical properties of plasma. Thermodynamic parameters, such as density *ρ* and enthalpy *h*, are derived from the molecular partition function, molecular mass, and other fundamental parameters based on the known number density of each plasma particle species. The transport parameters are computed using the established plasma equilibrium composition and the collision integral between particles. The Maxwell–Boltzmann distribution law facilitates the determination of the molecular population across various energy level states, including the electronic energy levels of atomic molecules and the electronic vibration–rotation energy levels of polyatomic molecules. Subsequently, radiation parameters of the plasma are further obtained by considering both the continuous radiation and the spectral line radiation emitted by each particle.

The equilibrium composition is calculated to ascertain the particle composition of the plasma, the number density of each particle species, and the distribution of particles across various energy level states. Determining the particle species present in the plasma and the chemical reactions among them is a prerequisite for establishing the equilibrium composition. On the basis of this foundation, the equilibrium composition model of arc plasma is constructed and solved. There are two main forms of plasma equilibrium composition models [1,2,3,4]. The first is developed by integrating Dalton’s law of partial pressures, stoichiometric equilibrium, and the law of conservation of charge with the Saha equation and the Guldberg–Waage equation [5]; the second involves solving for the minimum Gibbs free energy of the system to determine particle composition. Mathematically, these two approaches are equivalent in the gaseous state. The first method directly reflects the chemical process of plasma formation. Furthermore, since Saha and Guldberg–Waage are both equations, the abstract mathematical model is intuitive and concise. The Saha equation and the Guldberg–Waage equation reveal an explicit numerical relationship between the concentrations of reactants and products in the chemical reactions of plasma under thermodynamic equilibrium conditions. The ratio of the product of the concentrations of products to that of the reactants remains constant and is determined by the equilibrium temperature, the distribution coefficients of the reactants and products, and the reaction energy. Due to the varying nature of chemical reactions, the equilibrium coefficients of the Saha equation and the Guldberg–Waage equation can differ significantly, reaching differences on the order of 10^7^~10^9^. This substantial discrepancy contributes to the pronounced weak singularity in the nonlinear system of equations within the composition model [6].

The Gordon–McBride energy minimization method [7] is the most commonly used approach for solving the plasma equilibrium composition model. By defining a new Lagrange function by introducing the Lagrange multiplier *λ* (also referred to as the Lagrange Multiplier Method), the approach employs the Newton–Raphson iterative method with the steepest descent to identify the extremum near the initial value [8,9,10], thereby solving the model. In addition, Godin et al. [11,12] proposed the use of the concept of chemical basis to minimize the order of nonlinear equations to solve the equation. Frolov and Ivanov [13] used the modified Newton method of increasing the logarithm of unknowns to solve the model. Maria [14] and Zhong et al. [2] developed a new distribution function founded on the principle of maximum entropy and the theoretical framework of gas statistics, thereby bypassing the need to solve the Saha equation. While these methods contribute significantly to solving the equilibrium composition model, they have a notable drawback; they exhibit high sensitivity to the initial iteration value. If the selected initial value significantly deviates from the true value, the iterative process may converge to a local optimum, preventing the attainment of the global optimal solution.

In addition, Gleizes and Chervy et al. [9] used the Saha equation and the Guldberg–Waage equation to construct an SF_6_ plasma model at dual temperatures, and solved the composition model within 300~20,000 K and 0.1~1.6 Mpa. Wang and Rong et al. [8] investigated and solved the composition models of N_2_ and C(carbon)-H_2_O(water vapor) plasma at high temperature, and then calculated the physical properties and transport parameters of the plasma. Murphy et al. [15] calculated the physical properties and transport parameters of Ar, N_2_, O_2_, N_2_-Ar, and O_2_-Ar plasma within 300~30,000 K at atmospheric pressure. Saifutdinov [16] performed a series of numerical calculations, examining various modes of argon DC discharge under normal pressure in accordance with the unified model. The results of this study indicated that, contingent upon the cooling conditions of the electrodes, two forms of arc discharge could be obtained: one with a diffuse current spot and the other with a contracted current spot. In the present study, a plasma equilibrium composition model was established based on the Saha equation and the Guldberg–Waage equation for ionization and dissociation reactions. This model can be developed by incorporating Dalton’s law of partial pressures, the conservation of stoichiometry, and the law of conservation of charge. Fundamentally, the model constitutes a system of nonlinear equations characterized by a weakly singular Jacobian matrix, for which no universally applicable and effective solution method currently exists. Theoretically, existing solutions, such as Newton’s iterative method [17,18], the Levenberg–Marquardt Algorithm (LMA) [19,20,21], and the secant method [22,23], among others, can solve nonlinear systems of equations, but they are all more sensitive to the initial value of iteration. In other words, the premise for obtaining an accurate solution is a given initial value of iteration that is absolutely close to the “true value”.

In the present study, a plasma equilibrium composition model was constructed with a focus on analyzing its weak singularity, and two methods for solving the model were proposed. The main research content includes the following:

1. Based on the Saha and Guldberg–Waage equations, a plasma equilibrium composition model under local thermodynamic equilibrium and chemical equilibrium conditions was constructed by incorporating the stoichiometric number conservation equation, the charge conservation equation, and Dalton’s law of partial pressures. The model’s weak singularity was also analyzed to understand its mathematical and physical implications.
2. The equilibrium composition model is fundamentally a nonlinear system of equations characterized by a weakly singular Jacobian matrix, with its iterative solution being highly sensitive to initial values. To address this challenge, HLMA and the PV–LMA were proposed for solving the model. The principles, construction processes, and solution steps of these two algorithms were explored in detail to ensure a comprehensive understanding of their application and effectiveness in overcoming the sensitivity issue.
3. Taking N_2_, air and Mg−CO plasma as examples, HLMA and PV–LMA were used for calculations, followed by the analysis of the calculation process.
4. Finally, the feasibility of the HLMA and PV–LMA algorithms was verified, with a comparison of their advantages and disadvantages.

## 2. Equilibrium Composition Model and Weak Singularity Analysis

### 2.1. Equilibrium Composition Model

Plasma is a mixture of neutral particles produced by a series of chemical reactions [3]. For example, CO plasma undergoes chemical reactions such as decomposition CO=C+O and ionization CO=CO^+^ + e, C=C^+^ + e. It is, in essence, a mixture composed of an electron, C, O, C^+^, O^+^ and other particles. According to the conservation of stoichiometric number (for example, the stoichiometric ratio of CO_2_ is *n*_C_:*n*_O_ = 1:2), the ratio of the total density of two different elements is a constant, and its expression is:

(1) $$\sum_{m}l_{m,i}n_{m,i}:\sum_{m}l_{m,i}n_{m,1}=const$$

where *n_m,i_* is the total particle number density (cm^−3^) of the *i*-th element, *m* represents every particle in the plasma and *l_m,i_* is the stoichiometric number of the *i*-th element contained in the *m*-th particle of plasma.

According to Dalton’s law of partial pressures, the total pressure of a gas mixture is equal to the sum of the partial pressures of each individual gas component within it. The specific expression is:

(2) $$p=n_{e}k_{B}T+\sum_{i\neq e}^{m}n_{i}k_{B}T$$

where *p* is the plasma pressure (Pa); *n*_e_ is the electron number density; *k*_B_ is the Boltzmann constant (unit J/K, 1.38 × 10^−23^). *T* is the temperature of the plasma (K). According to the law of conservation of charge, plasma is electrically neutral on a macroscopic level. This is mathematically represented by Equation (3):

(3) $$\sum_{i}z_{i}n_{i}=0$$

where *z_i_* is the charged power of the *i*-th particle (including electrons, unit 1).

Under chemical equilibrium conditions, the chemical reactions in plasma can be categorized into two primary forms: ionization and dissociation. For ionization, the relationship between the number densities of reactants and products is described by the Saha equation, while the relationship between and the number density of the reactant products in the dissociation are expressed by the Guldberg–Waage equation. Their expressions are as follows:

(4) $$\frac{n_{e}n_{r+1}}{n_{r}}=\frac{2Q_{r+1}}{Q_{r}}{\left(\frac{2m_{e}\pi k_{B}T}{h^{2}}\right)}^{3/2}\exp\left(-\frac{E_{I,r+1}}{k_{B}T}\right)$$

(5) $$\frac{n_{A}n_{B}}{n_{AB}}=\frac{Q_{A}Q_{B}}{Q_{AB}}{\left(\frac{2\pi m_{A}m_{B}k_{B}T}{m_{AB}h^{2}}\right)}^{3/2}\exp\left(-\frac{E_{d}}{k_{B}T}\right)$$

where *h* is the Planck constant, J·s, 6.626 × 10^−34^. *Q_r_* and *Q_r+1_* are the partition functions of *A_r_* and *A_r+1_*, respectively. *Q*_AB_, *Q*_A_ and *Q*_B_ are the partition functions of molecules AB, A and B. *E_I, r+1_* and *E_d_* are the ionization energy of ionization and the dissociation energy of the dissociation, respectively, and the unit is eV or J/mol. *n*_r_ and *n*_r+1_ are the number densities of the *r* and *r + 1* cations of the atom. *n*_AB_, *n*_A_ and *n*_B_ are the p number densities of molecules AB, A and B. The calculation of the partition function can be guided by references such as *Molecular Spectroscopy and Molecular Structure: Diatomic Molecular Spectroscopy* [24] and other similar sources.

### 2.2. Weak Singularity Analysis of Model

In vector analysis, the term “Jacobian matrix” refers to a specific type of matrix that organizes the first partial derivatives of a function in a particular manner. A matrix is said to be “weakly singular” if, while being non-singular, its determinant values (also denoted by |***A***|) are close to zero, and if the ratio of its maximum and minimum values is often large. In practice, this matrix often exhibits singular characteristics, such as the fact that different initial values will yield different solutions, thereby resulting in multiple solutions.

The equilibrium composition model comprised Equations (1)–(3) multiple instances of (4), and (5), forming a nonlinear system of equations where the number density *n*_i_ of each particle species is the independent variable of the system of equations. In the charge conservation equation (Equation (3)), the particle charge *z_i_* = 0, −1, 1, 2,… (In the present study, *z*_i_ < 4). In the stoichiometric number conservation equation (Equation (1)), *l_m,i_* = 0, 1, 2,… and other integers. The order of magnitude of the *k*_B_·*T* value in the partial pressure equation (Equation (2)) was small, between 10^−21^ and 10^−19^ (J, kg·m^2^·s^−2^). The unit of the corresponding *p* was pressure (Pa, kg·m^−1^·s^−2^), and the atmospheric pressure was 101,325 Pa. According to the model, the independent variable of the nonlinear equations is the number density of each particle species, and their coefficients determine the specific form of the equations. According to common sense, the coefficients of the independent variables in Equation (1) are the sum of stoichiometric numbers, and their values are usually integers less than 30. The coefficient of the independent variable in Equation (2) is *k*_B_·*T*. When *T* = 300 K, its value is 1.9878 × 10^−30^; when *T* = 30,000 K, its value is 1.9878 × 10^−28^. In Equation (3), the coefficient of the independent variable is the charged energy of the particle, which is −1, 0, 1, 2…, where the integer is less than 8. From the above analysis, it can be seen that the coefficients of the same independent variable may differ by 1.9 × 10^−28^ times, which is significant.

It is evident that there is a significant disparity in the coefficients of these three equations, indicating the presence of weak singularity in the system. However, this issue can be mitigated by adjusting the values of *k*_B_·*T* and pressure *p* through unit conversion, such as converting the mass unit from kilograms (kg) to grams (g) and the length unit from meters (m) to centimeters (cm).

**Figure 1:**
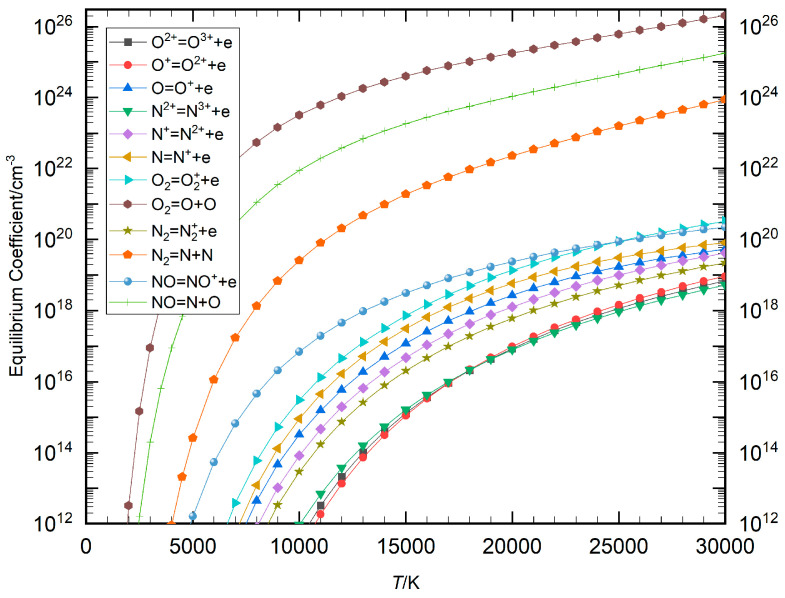
Change curve of each equilibrium coefficient with temperature in the air plasma.

**Figure 2:**
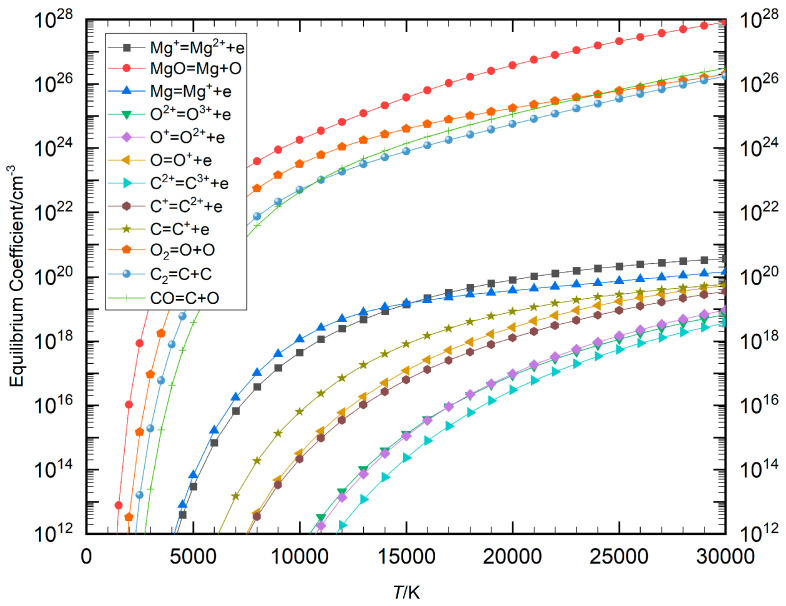
Change curve of each equilibrium coefficient with temperature in Mg−CO plasma.

In the Saha and Guldberg–Waage equations, there was a significant disparity in the values on the right-hand side of the chemical reaction equations (Equations (4) and (5)), which is due to varying chemical reaction energies. This value, referred to as the equilibrium coefficient in the present study, illustrates the distinct energy requirements for different reactions. Using air plasma and a Mg−CO mixture plasma as examples, the weak singularity of the equilibrium composition model can be analyzed by calculating the temperature-dependent change curves of the equilibrium coefficients for each chemical reaction, as shown in Figure 1 and Figure 2. The figures indicate that there was a considerable gap between the equilibrium coefficients for dissociation and ionization reactions. The magnitude of these coefficients was found to be diminished, and as a consequence, they were not incorporated within the figures. However, as the temperature increased, the equilibrium coefficients became larger, with a more pronounced rate of change observed in the lower temperature range.

**Figure 3:**
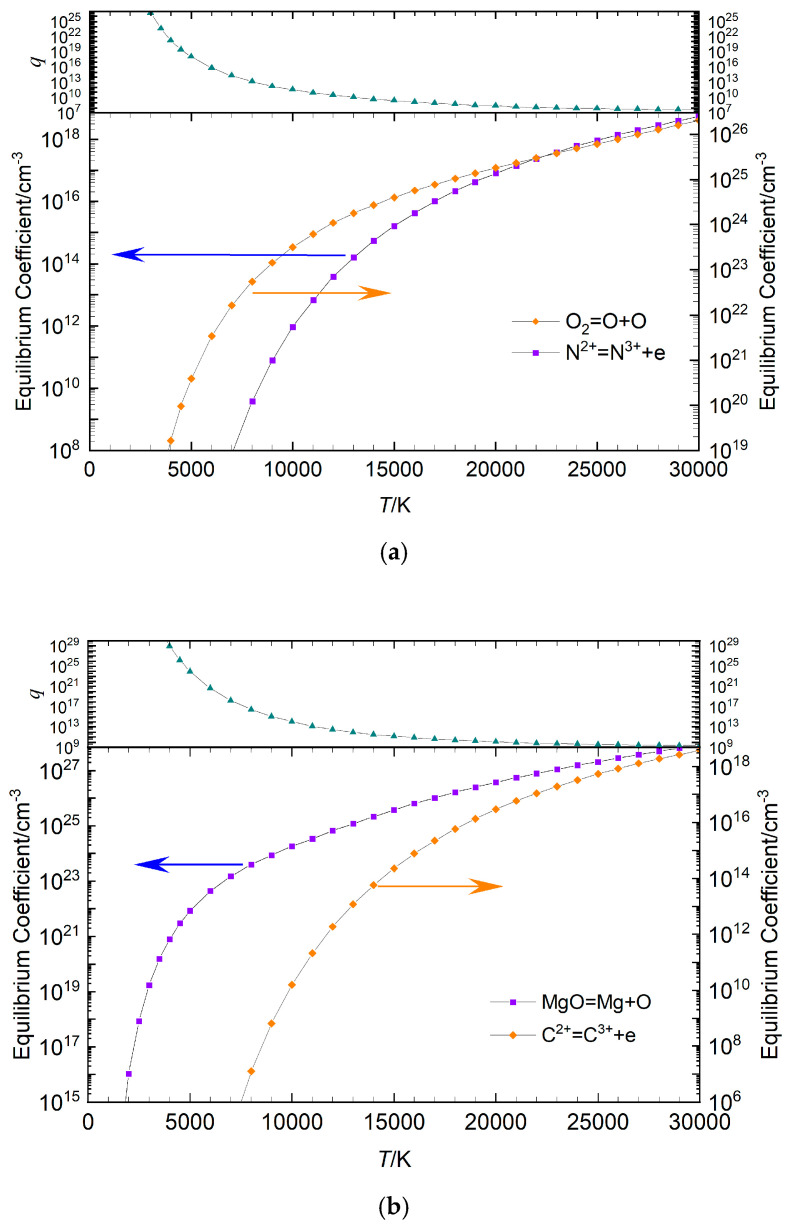
The change curves of the maximum and minimum equilibrium coefficients in the plasma and their ratio *q* with temperature: (**a**) air plasma, (**b**) Mg−CO mixture plasma.

The comparison curves of the maximum and minimum equilibrium coefficients for air plasma and Mg−CO mixture plasma as functions of temperature are presented in Figure 3, alongside the temperature-dependent change curve of their ratio *q*. The data show that in air plasma, the equilibrium coefficient for the reaction O_2_⇌O+O was the largest, while that for the reaction coefficients of N^2+^⇌N^3+^+*e* was the smallest. The overall gap between these coefficients was significant, with the disparity more pronounced at lower temperatures than at higher temperatures. The trend of Mg−CO plasma was the same as that of air, where the chemical equations for the maximum and minimum equilibrium coefficients were MgO ⇌ Mg + O and C^2+^⇌C^3+^ + *e*, respectively. The scale of their coefficients indicated *q* = 1 × 10^9^ at 30,000 K and *q* = 1 × 10^29^ at 4000 K. As the temperature decreased, the *q* value became greater. As demonstrated in Figure 1, Figure 2 and Figure 3, the equilibrium coefficients of disparate chemical reactions exhibit considerable variation at constant temperature. In terms of reaction type, the equilibrium coefficient of the dissociation reaction exhibits a significantly higher value in comparison to the equilibrium coefficient of the ionization reaction at low relative temperature. As illustrated in Figure 3, at a temperature of 5000 K, the ratio of the maximum equilibrium coefficient to the minimum equilibrium coefficient in the air plasma is approximately 10^28^, and in the Mg−CO mixture plasma, it is approximately 10^29^. It was determined that the equation coefficients resulting from the equilibrium coefficients of chemical reactions exhibit significant variation.

The iterative method is widely utilized for solving nonlinear systems of equations. The effectiveness of this approach relies heavily on the chosen iterative function, as it determines whether the process converges or diverges. The iterative function for a nonlinear system of equations incorporates the Jacobian matrix, which plays a crucial role in guiding the solution process. The calculation methods for the Jacobian matrix are provided in Equations (6) and (7).

(6) $$J=\left|\frac{\partial \left(f_{1},\cdots f_{n}\right)}{\partial \left(x_{1},\cdots ,x_{n}\right)}\right|=\left|\begin{array}{ccc} \frac{\partial f_{1}}{\partial x_{1}} & \cdots & \frac{\partial f_{1}}{\partial x_{n}}\\ \vdots & \ddots & \vdots \\ \frac{\partial f_{n}}{\partial x_{1}} & \cdots & \frac{\partial f_{n}}{\partial x_{n}} \end{array}\right|$$

(7) $$\frac{\partial f_{i}}{\partial x_{j}}=\frac{f_{i}\left(x_{1},\cdots ,x_{j}+dx,\cdots ,x_{n}\right)-f_{i}\left(x_{1},\cdots ,x_{j},\cdots ,x_{n}\right)}{dx}$$

In the calculations herein, if *x_j_* = zero, then *dx* = 1 × 10^−5^; otherwise, *dx* = 1 × 10^−5^ × *x_j_*. Evidently, when there is a significant gap in the equilibrium coefficients of a system of equations at a given temperature, the disparity in the elements of the Jacobian matrix ***J*** is also substantial. This condition results in the matrix being weakly singular.

## 3. Solution Method

Developing an iterative solution method for nonlinear equations with minimal dependence on initial values is critical for the study of plasma equilibrium composition models [25,26,27], and is a primary focus of the present research. The homotopy algorithm, known for its flexibility with initial values, has emerged as an effective approach for solving nonlinear systems of equations [28,29,30]. However, for systems characterized by a weakly singular Jacobian matrix, constructing auxiliary equations and determining the homotopy factor sequence [*t_k_*] remain central challenges in research. LMA [20,31] is an iterative solution method for nonlinear systems of equations that incorporates an adaptive step size. This method is applicable to both singular and non-singular systems, providing the advantage of high computational speed. Additionally, LMA functions as a hybrid approach that combines elements of the Gauss–Newton algorithm and the steepest descent method. The use of an adaptive step size in LMA enhances the iterative process by increasing its capability to avoid local optima, thereby improving the likelihood of converging to a global solution.

### 3.1. Homotopy Levenberg−Marquardt Algorithm (HLMA)

By integrating the strengths of the homotopy algorithm and the LMA, a new algorithm with reduced sensitivity to initial values is proposed for solving the plasma equilibrium composition model, referred to as the HLMA. In this approach, the homotopy method is used to generate initial values for the LMA, which subsequently performs a rapid solution of the homotopy equations. The core concept of HLMA is “the provision of a reasonable initial value for LMA solution of the *n*-th homotopy equations through *(n-1)*-th of homotopy calculation”. This effective combination of the homotopy method and LMA enables the resolution of the plasma equilibrium composition model with improved robustness. Since the initial value in the iterative solution of a nonlinear system of equations directly influences the accuracy of the results, its selection has historically been a challenging aspect of these solutions. The homotopy method addresses this challenge by constructing suitable auxiliary equations, allowing the entire solution process for the nonlinear system to proceed without concerns regarding the sensitivity to initial values.

The key step in the homotopy method is the construction of auxiliary equations. This involves creating a new mapping that matches the dimension of the original system of equations to facilitate the solution of a nonlinear system. The basic form of the homotopy system of equations is expressed as:

(8) $$H\left(x,t\right)=tF\left(x\right)+\left(1-t\right)G\left(x\right)=0$$

where ***H***(***x***, *t*) represents the homotopy equations; ***F***(***x***) is the original equation to be solved; and ***G***(***x***) represents the auxiliary equations. When *t* = 0, the homotopy equations reduce to the auxiliary equations, and when *t* = 1, the homotopy equations become equivalent to the original equations. Due to the large difference between the equilibrium coefficients on the right side of the Saha and Guldberg–Waage equations (up to 10^7^~10^9^ orders of magnitude), the plasma equilibrium composition model exemplifies a nonlinear system with a weakly singular Jacobian matrix, making it more challenging to solve. This issue is thoroughly discussed in Section 2.2. Under the premise of obtaining a reliable initial iteration value, LMA can achieve quick calculation of more accurate results. The construction of homotopy equations that can be accurately solved is a crucial prerequisite for the effective application of the homotopy method in solving nonlinear systems of equations.

#### 3.1.1. Construction of the Homotopy Equations

Constructing homotopy equations involves defining an auxiliary equation and determining the arrangement of the homotopy factor *t*. The auxiliary equations must meet two main criteria. First, they should have the same dimension as the original system of equations; second, they should have a solution that either meets the necessary accuracy requirements or can easily obtained. There are various methods for constructing homotopy equations: for example, fixed-point homotopy equations ***G***(***x***) = ***x*** − ***x***_0_, ***H***(***x***, *t*) = *t**F***(***x***) + (1 − *t*)(***x*** − ***x***_0_). Convex homotopy equations ***G***(*x*) = ***A***(***x*** − ***x***_0_), ***H***(***x***, *t*) = *t**F***(***x***) + (1 − *t*)***A***(***x*** − ***x***_0_), where ***A*** is a non-singular square matrix. Newton’s homotopy equations, ***G***(*x*) = ***F***(*x*) − ***F***(*x*_0_), ***H***(*x*, *t*) = *t**F***(*x*) + (1 − *t*)***A***(*x* − *x*_0_), where ***A*** is a non−singular square matrix. Newton’s homotopy equations, ***G***(***x***) = ***F***(***x***) − ***F***(***x***_0_), ***H***(***x***, *t*) = ***F***(***x***) + (*t* − 1)***F***(***x***_0_). Notably, when ***F****’*(***x***) is weakly singular, performing calculations using these methods or conventional homotopy numerical solution techniques becomes challenging.

In the plasma equilibrium composition model, temperature and pressure are critical factors influencing the composition distribution. Generally, under a given pressure, the higher the temperature, the greater the electron number density in the ionized state, meaning the ionization ratio *n*_e_/Σ*_i_*(*n_i_*) increases. Under high−temperature conditions, the number density of the plasma equilibrium composition can be accurately determined based on this assumption. This assumption can be directly utilized as the solution for the model or as the initial value for iterative calculations. For instance, in the case of air plasma at a temperature of 30,000 K for both large particles and electrons, using this hypothesis as the initial value for an iterative solution yields an accuracy of ***eps*** = Σ*_i_*(*f_i_*(*x*))^2^ = 1.16 × 10^−28^. Therefore, the high-temperature composition equations are used as auxiliary equations in constructing the homotopy equations:

(9) $$H_{T}\left(x,t\right)=tF_{T}\left(x\right)+\left(1-t\right)F_{T_{\max}}\left(x\right)=0\begin{array}{cc} & t\in \left[0,1\right] \end{array}$$

The auxiliary equations ***F***_Tmax_(***x***) = 0 represent the composition equations at a specified high temperature *T*_max_. These auxiliary equations are solved using the LMA, with an initial value that is numerically calculated and consists only of electron and atomic cations with the highest valence state. If the solution does not achieve the required accuracy, the temperature will incrementally increase until the desired accuracy is reached. In addition, ***F***_Tmax_(***x***) = 0 can also represent a composition equation where the temperature is higher than the solution temperature and meets the accuracy requirement.

#### 3.1.2. Homotopy Sequence Arranged Proportionally

The numerical solutions of traditional homotopy algorithms include the homotopy continuation method [32,33,34], the parametric homotopy approach [35], and the homotopy perturbation method [36,37]. The ultimate goal of these methods is to construct a homotopy sequence [*t*_k_] within the interval [0, 1]: 0 = *t*_0_ < *t*_1_ < *t*_2_ < ⋯ < *t*_N_ = 1. After continuous advancement of calculations, the solution at *t* = 1 was finally obtained. The calculation expression of the homotopy equations at *t_k_* is denoted in Equation (10).

(10) $$H_{T}\left(x,t_{k}\right)=t_{k}F_{T}\left(x\right)+\left(1-t_{k}\right)F_{T_{\max}}\left(x\right)=0,k=1,2,\cdots ,N$$

The construction of the homotopy sequence [*t_k_*] and the determination of the auxiliary equations ***F***_Tmax_(***x***) = 0 that can be accurately solved are crucial for the success of the entire homotopy process. The earlier discussion covered the formulation of ***F***_Tmax_(***x***) = 0 and obtaining its precise solution. From the analysis of the equilibrium composition model, it can be determined that the factor that causes the weak singularity of the system of equations lies in the function constructed by the Saha and Guldberg–Waage equations. In the present study, the largest singularity coefficients *S*_T,max_ and *S* in the ***F***_Tmax_(***x***) = 0 and ***F***(***x***) = 0 equations were selected as homotopy reference data, and the singularity coefficient *R*_max_ = *S*_T,max_/*S* was determined. In principle, a larger number of homotopy steps ***N*** is better. In the present study, the larger number of homotopy steps is *N* = (*T*_max_ − *T*)/50.

Proportional sorting is adopted. The scale coefficient *q* and the homotopy coefficient *t_k_* are denoted in Equations (11) and (12), respectively.

(11) q=Rmax1N

(12) $$t_{k}=\frac{R_{max}-q^{N-k}}{R_{max}}$$

#### 3.1.3. HLMA Calculation Steps

**Figure 4:**
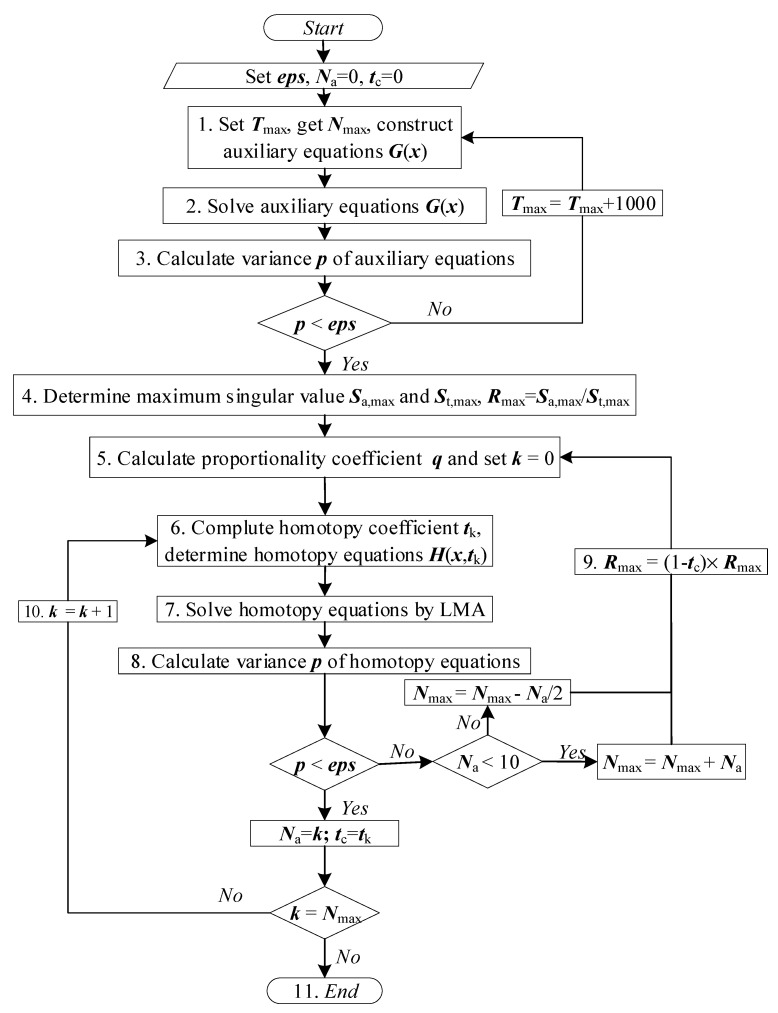
Calculation flow chart of the HLMA algorithm.

The calculation process of the HLMA algorithm is shown in Figure 4. The main process of solving the plasma equilibrium composition model is divided into two steps. First, the auxiliary equations need to be determined; these equations are composition equations at a high temperature (such as 30,000 K) or a temperature higher than the solution temperature, where the required accuracy is met. This step provides a reliable starting point for the iterative process. Second, the homotopy sequence [*t*_k_] must be established to ensure continuous and smooth progression of the homotopy. In the present study, the largest singular coefficients *S*_T,max_ and *S* in the equations ***F***_T,max_(**x**) = 0 and ***F***(***x***) = 0 were selected as the homotopy reference data, and the singular coefficient *R*_max_ = *S_T_*_,max_/S was determined. The homotopy sequence was determined by the arrangement of proportional series.

The calculation steps of the solution of the plasma equilibrium composition model with the HLMA algorithm are as follows.

1. Determine the limit operating condition *T*_max_, and the number of homotopy steps *N*_max_; set the calculation accuracy *eps* < 1 × 10^−15^; and construct the auxiliary equations.
2. Assume that the plasma contains only electrons and atomic cations with the highest valence. Construct and solve a system of *N*_e_ + 1 linear equations based on the law of conservation of charge, Dalton’s law of partial pressures, and the constancy of the total atomic number density ratio to obtain an accurate solution or reasonable initial iteration value.
3. Solve the auxiliary system of equations and its square difference *p* to determine whether *p* < *eps*. If it is established, proceed to Step 4; otherwise, *T*_max_ = *T*_max_ + 1000, and return to Step 1.
4. Find the maximum singular value *S*_max_ of the auxiliary system of equations.
5. Calculate the scale coefficient, and set *k* = 0.
6. Calculate the homotopy coefficient and determine the homotopy equations.
7. Solve the homotopy equations with LMA and calculate the square difference *p* of the homotopy system of equations.
8. Determine whether *p* < *eps*. If it is not established, then proceed to Step 9; otherwise, record the completed homotopy number *N*_a_ = *k* and the homotopy control factor *t*_c_ = *t_k_*_−1_, and proceed to Step 10.
9. Determine whether *N*_a_ < 10. If it is established, set *N*_max_ = 2 × *N*_max_; otherwise, *N*_max_ = *N*_max_, and return to Step 5.
10. Determine whether *k* = *N*_max_. If it is not established, *k* = *k* + 1 and return to Step 6; otherwise, proceed to Step 11.
11. The process concludes.

### 3.2. Parameter Variation Levenberg−Marquardt Algorithm (PV–LMA)

#### 3.2.1. The Principle of PV–LMA

The parameter variation shares similarities with the homotopy algorithm in that both solve a series of parametric equations ***H***(***x***, *t*) = 0 under the conditions of auxiliary functions and known solutions, ultimately achieving the solution of ***H***(***x***, 0) = ***F***(***x***) = 0. In addition, both algorithms require the Jacobian matrix ***J*** of the parametric equation ***H***(***x***, *t*) = 0 to be non-singular. However, as indicated by the aforementioned discussion, addressing the ***J***−singularity of nonlinear equations in the plasma equilibrium composition model is challenging when using only the homotopy method or PVM independently. To overcome the issue of singular nonlinearity, the PVM algorithm is employed by selecting a suitable approach for handling singularities and incorporating the PVM framework to solve the parametric equation effectively. LMA is highlighted as an exceptional method for addressing singular nonlinear equations. Therefore, if PVM can provide a reliable iterative initial value for LMA, the PV equation is accurately solved with LMA.

The form of the Parameter Variation (PV) equation for the PV–LMA is expressed as:

(13) $$H\left(x,t\right)=tF\left(x\right)+\left(1-t\right)F\left(x_{0}\right)=0$$

where ***x***_0_ is the given initial value. First, ***F***(***x***_0_) is calculated given the initial value to obtain a one-dimensional array [*b*]. Then, the above PV equation becomes Equation (14).

(14) $$H\left(x,t\right)=tF\left(x\right)+\left(1-t\right)b=0$$

Another important part of solving the system of equations is the selection of the factor sequence [*t_k_*]. In PV–LMA, arithmetic series are selected for the arrangement. Then, the above formula becomes a series of Equation (15). This method is similar to HLMA, where multiple equations need to be solved in order to achieve the solution of the final equation.

(15) $$H\left(x,t_{k}\right)=t_{k}F\left(x\right)+\left(1-t_{k}\right)b=0$$

#### 3.2.2. PV–LMA Calculation Steps

**Figure 5:**
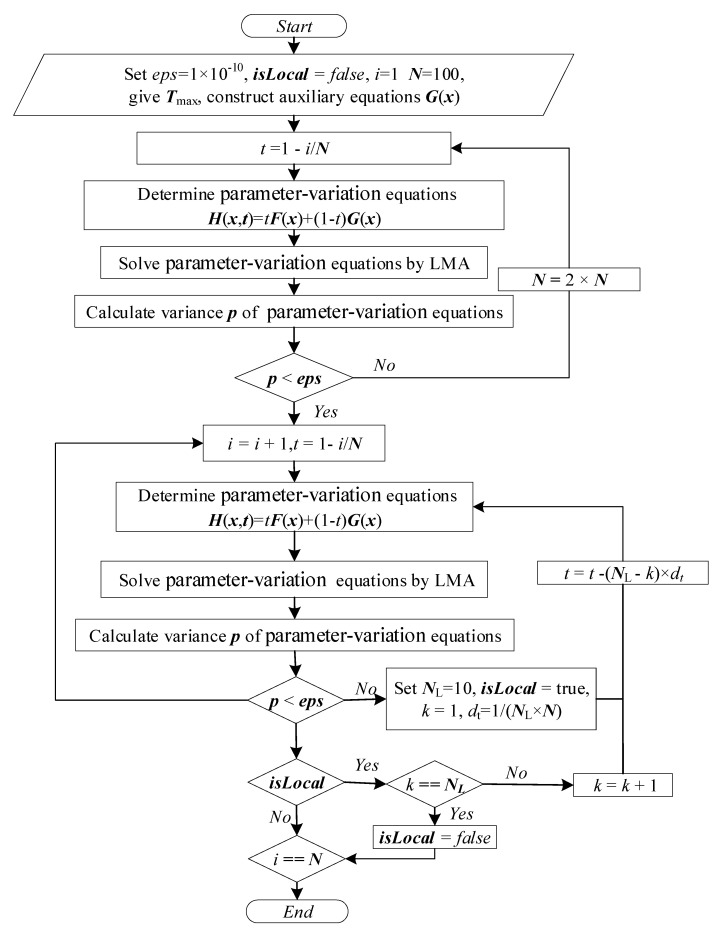
Calculation flow chart of PV–LMA algorithm.

The calculation process of the PV–LMA is illustrated in Figure 5. The core of this process involves solving each PV equation and refining the solution locally in case of failure. The entire calculation is structured into three main parts: determining the number of calculations *N*, solving the PV equation, and refining the solution of the PV equation locally. The detailed calculation steps are as follows:

1. Set the calculation accuracy *eps*, the parameter variation factor *i*, the number of variations ***N***, indicator for localized solution process ***isLocal***, and known solution temperature ***T***_max_. *eps* is used to set the solution accuracy to ensure that the solution of the PV equation meets the calculation requirements. The variation factor and the number of variations are used to determine the coefficient of variation. ***isLocal*** identifies whether it is a local calculation process, and the solution ***x***_0_ of ***T***_max_ and high temperature is used to construct an auxiliary array [*b*].
2. Determine the variation coefficient *t* = 1 − *i*/***N***.
3. Construct the PV system of equations ***H***(***x***, *t*) based on the variation coefficient T.
4. Solve the PV system of equations ***H***(***x***, *t*) with LMA algorithm.
5. Calculate the equation *p* of the PV system of equations ***H***(***x***, *t*).
6. Determine whether *p* < eps. If it is established, then proceed to Step 7; otherwise, ***N*** = 2 × ***N*** and return to Step 2.
7. Set *i* = *i* + 1, calculate the variation coefficient *t*, *t* = 1 − *i*/***N***.
8. Construct the PV system of equations H(x,t) based on the variation coefficient T.
9. Solve the PV system of equations ***H***(***x***,*t*) with the LMA algorithm.
10. Calculate the equation *p* of the PV system of equations ***H***(***x***,*t*).
11. Determine whether *p* < eps. If it is established, then proceed to Step 15; otherwise, return to Step 12.
12. Set ***N***_L_ = 10, ***isLocal = true***, *k* = 1, and calculate *d*_t_ = 1/(***N***_L_ × ***N***).
13. Calculate the compilation parameter *t*, *t* = *t* − (***N***_L_ − *k*) × *d*_t_.
14. Perform Steps 8, 9, and 10.
15. Determine whether ***isLocal = true***. If it is established, then proceed to Step 16; otherwise, proceed to Step 17.
16. Set *k* = *k* + 1, and execute steps 13 and 14.
17. Determine whether *i* = ***N***. If it is established, then proceed to Step 18; otherwise, return to Step 7.
18. The process concludes.

### 3.3. Comparison of PV–LMA and HLMA Algorithms

Although PV–LMA was developed based on HLMA, there are obvious differences and similarities between the two. Table 1 provides a comparison between HLMA and PV–LMA. Their similarities and differences are analyzed in terms of the equation structure and calculation process.

From the structural perspective of homotopy and PV equations, the HLMA auxiliary equations represent the plasma equilibrium composition at a specified temperature, whereas the PV–LMA auxiliary equations are conceptualized as a one-dimensional array [*b*]. This distinction implies that when there is a substantial difference between the auxiliary temperature and the solution temperature, the HLMA algorithm is preferable. The homotopy factors, distributed in equal proportions, facilitate the adjustment of the balance between the auxiliary equations and the solution equation within the homotopy framework. Conversely, when the temperature difference is minimal, the PV–LMA algorithm is more advantageous due to its alignment with small variations in temperature.

From the selection of the control factor sequence, the homotopy factor *t_k_* of the HLMA algorithm was determined using the arrangement of proportional sequence. As the number of homotopy iterations increased, the difference between successive homotopy factors, *Δt* = *t*_k_ − *t*_k−1_, gradually diminished. When the homotopy equations were significantly different from the solution equation, the precise solution of the auxiliary equations more easily met the requirements for the initial iterative value of the homotopy equations. As the homotopy equations approached the solution equation, the difference between consecutive homotopy equations became smaller, allowing for successful solution convergence. In the context of the PV–LMA algorithm, where the parametric factor *t_k_* (analogous to the homotopy factor in homotopy equations) is distributed arithmetically, solutions are theoretically attainable for any given initial value. Nonetheless, considering computational costs and the variation in the plasma density distribution curve with temperature, using the number density at higher temperatures as the initial value is undeniably the most efficient approach.

From a procedural standpoint, in the HLMA method, the equilibrium composition equation can be computed twice within each iteration (solving equations ***F***(***x***) and auxiliary equations ***F***_Tmax_(***x***)). In PV–LMA, however, only equation ***F***(***x***) needs to be solved, which reduces the amount of calculation by half and requires fewer computing resources. Regardless of whether the HLMA homotopy equations or the PV–LMA equation is involved, both approaches leverage the LMA, known for its high computational efficiency, to achieve solutions.

## 4. Calculation Examples

In order to verify the effectiveness of HLMA and PV–LMA, three examples were selected for the calculation, namely nitrogen plasma (*N*_e_ = 1), air (N_2_ 79%−O_2_ 21%) plasma (*N*_e_ = 2) and fused magnesia arc (Mg50%−CO 50%) plasma (*N*_e_ = 3). The specified calculation temperatures *T* were 6000 K, 25,000 K and 10,000 K, and the values of the auxiliary temperature *T*_max_ were 7000 K, 30,000 K and 12,000 K, respectively. These results demonstrate that the auxiliary temperatures can range from high temperatures (e.g., 30,000 K) to intermediate values (e.g., 7000 K and 12,000 K), each yielding precise solutions.

### 4.1. Nitrogen Plasma

The particle composition of the N_2_ plasma is listed in Table 2, including two neutral particles, four cations, and electrons. The calculation requirements are solution temperature *T* = 10,000 K, auxiliary temperature *T*_max_ = 12,000 K, environmental pressure *p* = 1 *atm*, and calculation accuracy *eps* < 1 × 10^−10^. Nitrogen plasma comprises only the element N, resulting in a relatively simple particle composition. This implies that its equilibrium composition model does not include an equation for stoichiometric number conservation. The plasma composition encompasses five chemical reactions, consisting of one decomposition reaction and four ionization reactions. The degree of ionization for the nitrogen atom in this context is 3. Table 3 outlines these chemical reactions, highlighting the fundamental processes that contribute to the plasma’s equilibrium state.

**Figure 6:**
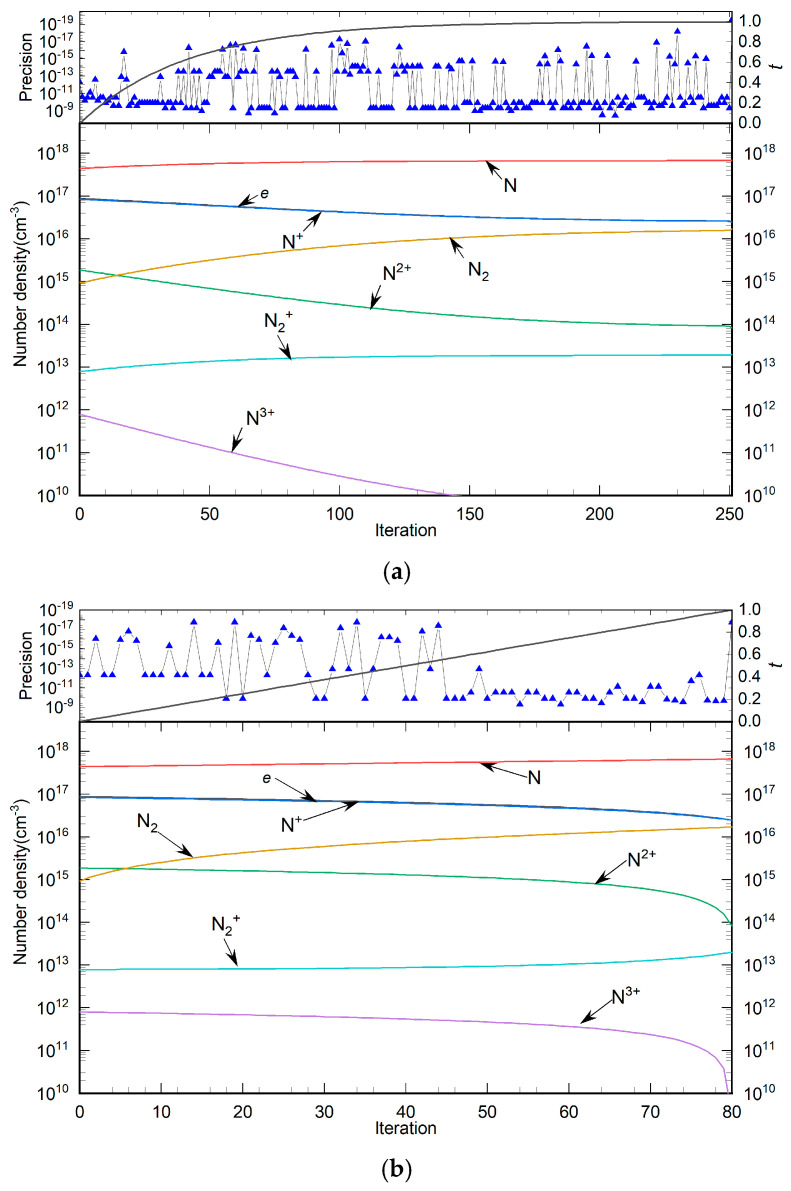
The change curves of homotopy factor, accuracy and particle number density of nitrogen plasma with the number of homotopy steps in the two algorithms: (**a**) HLMA, (**b**) PV–LMA.

Table 4 shows the solution of the auxiliary and the solving equations for N_2_ plasma. Figure 6 presents the variation curves for particle number density, homotopy factor, and calculation accuracy for each particle across the number of homotopy steps under the HLMA and PV–LMA algorithms. The results align with expectations, with the HLMA algorithm requiring 250 iterations compared to the 80 iterations in the PV–LMA method, demonstrating PV–LMA’s superior computational efficiency. Significant variation in the number density of N^3+^ indicates that the temperature interval corresponds to the initiation of the reaction N^2+^⇌N^3 +^ + *e*, and the curves of all seven particles involved confirm the presence of each chemical reaction within the temperature range. Continuous changes in both the number density and homotopy factor are observed, with each calculation meeting accuracy requirements and the final solution transitioning smoothly from the initial estimate, consistent with the homotopy method’s numerical approximation. However, the PV–LMA algorithm shows a pronounced change in particle number density near the end of the process, suggesting that HLMA has clear advantages in ensuring the continuity of the calculation.

### 4.2. Air Plasma

The neutral molecular components of the plasma are nitrogen N_2_ (79%) and oxygen O_2_ (21%). Namely, *N*_e_ = 2; temperature *T* = 6000 K; total pressure *p* = 1 *atm*; and calculation accuracy *eps* < 1 × 10^−15^. The conditions of auxiliary equations are *T*_max_ = 7000 K and the calculation accuracy *eps* < 1 × 10^−15^. The particles involved in the air plasma included five kinds of neutral particles, nine kinds of cations and electrons. These particles were connected by twelve chemical reactions, including three dissociation reactions and nine ionization reactions. The particle compositions of the air plasma and the involved chemical reactions are listed in Table 5 and Table 6, respectively. These particles and chemical reactions will play a leading role in the subsequent calculation of equilibrium components, thermodynamic parameters, transport parameters, and net radiation coefficient. Here, only the third-order ionization of N and O is considered, because their ionization energy of fourth order is higher. The N^4+^ and O^4+^ particles have not been considered herein given their high precipitation temperature (higher than 20,000 K).

**Figure 7:**
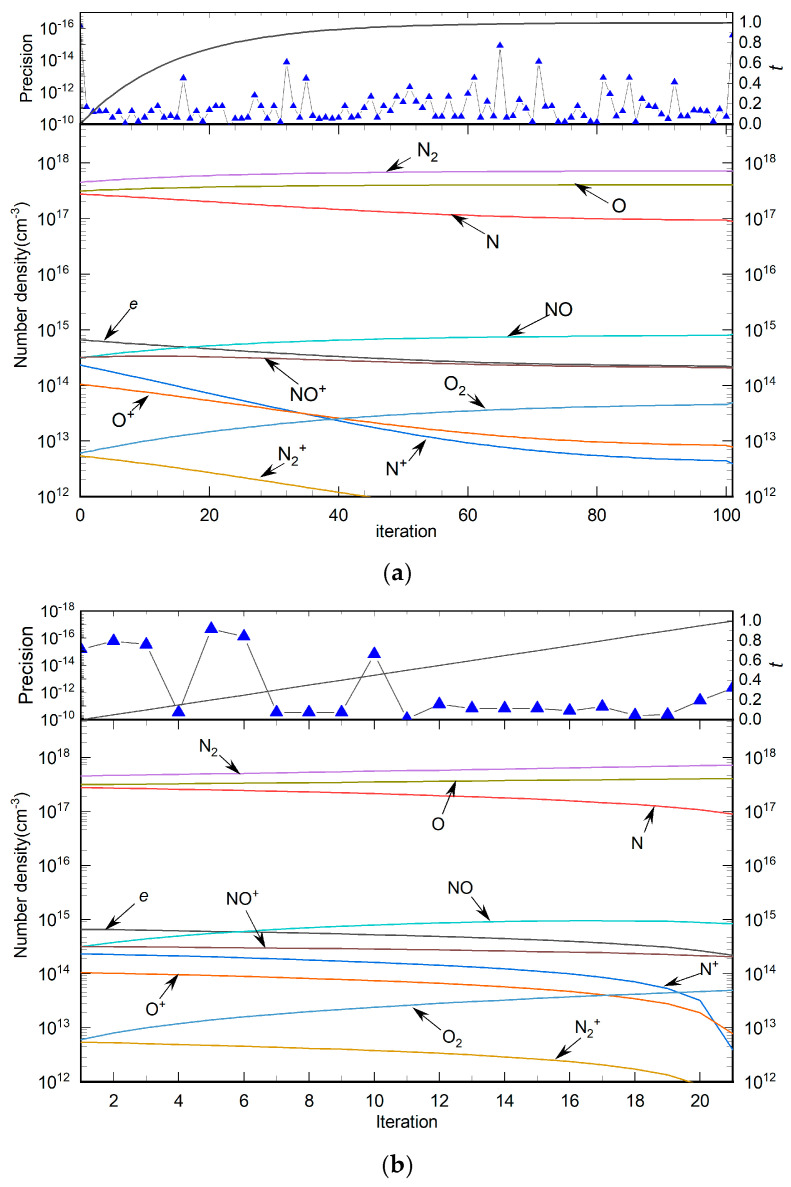
The change curves of homotopy factor, accuracy and particle number density of air plasma with the number of homotopy steps in the two algorithms: (**a**) HLMA, (**b**) PV–LMA.

Table 7 lists the number density of each particle species of the plasma at temperatures *T* = 7000 K, *T* = 6000 K, and *p* = 1 *atm*. The calculation results of HLMA and PV–LMA were close, but with only a slight difference below the order of 10^4^. Therefore, they have not been listed separately in the table. Figure 7 show the change curves of homotopy factor, accuracy, and particle number density with the number of homotopy steps in HLMA and PV–LMA algorithms. The particle number density of N^3+^ and O^3+^ is not given in the figure and table. This is because the chemical reactions N^2+^⇌N^3+^ + *e* and O⇌O^+^ + *e* did not occur (or the reaction intensity was low) in this temperature interval. The solution accuracies of the equilibrium composition model at the two temperatures were 6.46 × 10^−23^ and 2.65 × 10^−16^, respectively, both of which were high.

The figure shows that HLMA and PV–LMA underwent 100 and 20 iterations, respectively, highlighting the higher computational efficiency of PV–LMA compared to HLMA. In the left figure, the homotopy control factor *t_k_* progressed from 0 to 1, with the gap between neighboring control factors narrowing as the process advanced, thereby ensuring the continuity of the homotopy process. Throughout the calculations, each variable transitioned smoothly from its initial value to the final solution, maintaining the correct iterative direction and avoiding local optimization traps. In HLMA, if local optimization occurs, adjusting the step size of the homotopy control factor enables the LMA to escape local optima by dynamically modifying the trust region’s range. This integration of homotopy with the LMA algorithm is advantageous for solving nonlinear problems, as it secures the correct optimization path while smoothly bypassing local optima. Moreover, when selecting an auxiliary function, a higher temperature than the current solved temperature at the same pressure is chosen. The right figure illustrates that the control factor *t* changes linearly with iteration steps, with significant changes in particle number density occurring late in the PV–LMA iterations, contrary to HLMA, where density changes are more prominent early in the process. This indicates that PV–LMA is less effective than HLMA in ensuring the continuity of iterations.

### 4.3. Mg50%−CO 50% Plasma

The mixed Mg50%−CO 50% plasma contains three elements (*N*_e_ = 3): the calculated temperature *T* = 25,000 K; the auxiliary temperature *T*_max_ = 30,000 K; and the plasma pressure *p* = 1 *atm*. There were a total of nineteen particles (eight neutral particles and twelve cations) and fifteen chemical reactions (five dissociation reactions and ten ionization reactions) in the mixed plasma. Table 8 and Table 9 list the particles in the plasma and the types of chemical reactions involved, respectively. Here, the ionization degree of metal Mg particles was 2, and the ionization degree of C and O was 3. This was also ignored due to the higher energy required for further ionization and the higher precipitation temperature of particles.

Table 10 lists the number density of each particle species in the plasma at the auxiliary temperature and solution temperature. As shown, the solution accuracy of the equilibrium composition model at both temperatures was less than 1 × 10^−15^. There was a large gap in the transitional number density of polyatomic molecules from 30,000 K to 25,000 K. For example, the value of O2 transitioned from 7.59 × 10^−35^ to 12.7, and CO transitioned from 1.27 × 10^−35^ to 5.14. The gap between other polyatomic molecules was also around 1030. Due to the decrease in temperature, the total number density gradually increased at the constant pressure. Here, the number density of C, O, C^+^, O^+^, O^2+^, O^2+^, Mg and Mg^+^ changed significantly, while the changes in Mg^2+^, C^3+^ and O^3+^ were less significant. Notably, Table 10 shows that the calculation accuracy of HLMA was 1.1 × 10^−16^, while the calculation accuracy of PV–LMA was 8.7 × 10^−16^; both are of the same order of magnitude.

**Figure 8:**
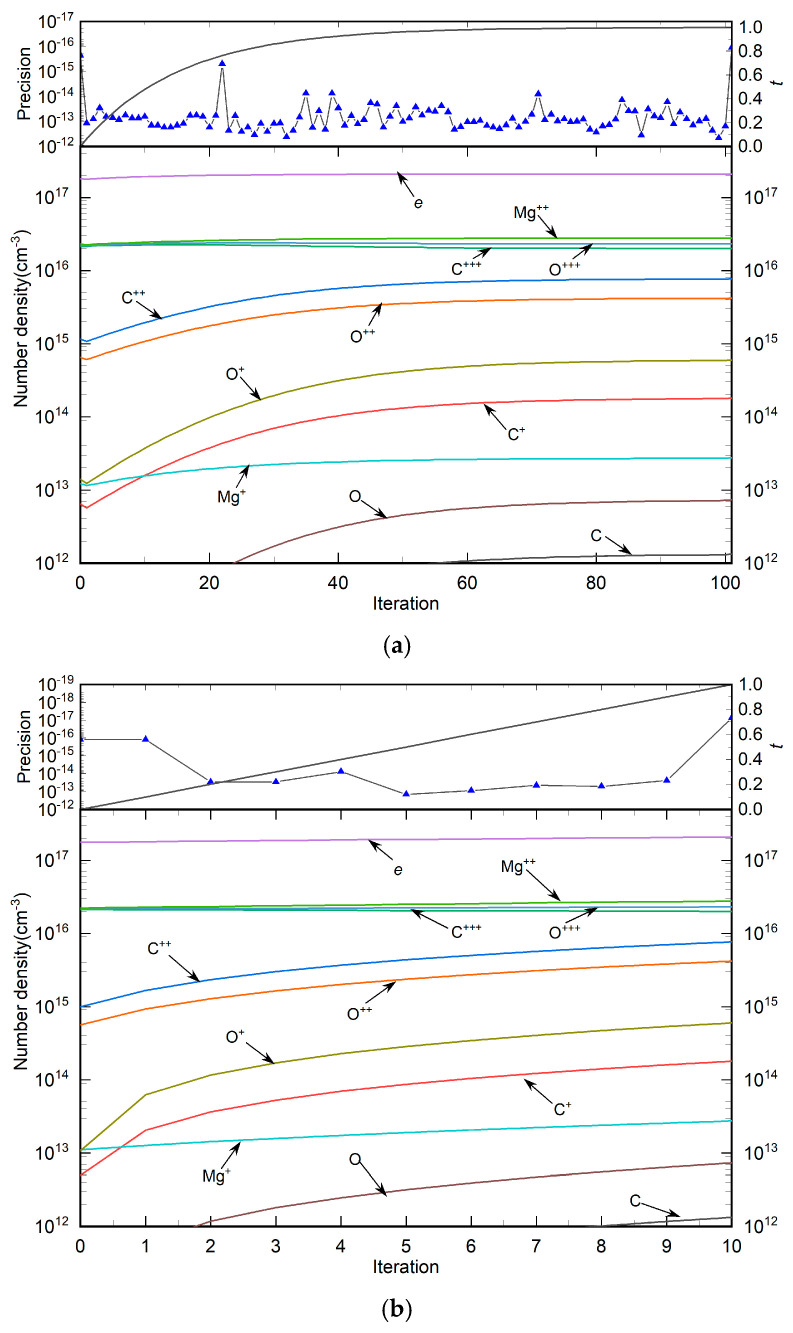
The change curves of plasma homotopy factor, accuracy and particle number density of the mixed Mg50%−CO% 50% plasma with the number of homotopy steps in the two algorithms: (**a**) HLMA, (**b**) PV–LMA.

Figure 8 shows the change curves of the homotopy factor *t_k_*, accuracy *eps*, and particle number density with the number of homotopy steps in solving the mixed Mg50%−CO 50% plasma with HLMA and PV–LMA algorithms, respectively. In this calculation, the HLMA algorithm performed 100 homotopy calculations, whereas the PV–LMA algorithm completed only 10 iterations. Both algorithms achieved a calculation accuracy of less than 1 × 10^−10^. Their final calculation accuracies were 1.11 × 10^−16^ and 7.78 × 10^−18^, respectively.

It can been observed from the figure and table that at higher temperatures (30,000 K), the dominant particles in the plasma were atomic cations with the highest valences (Mg^2+^, C^3+^, and O^3+^) along with electrons, collectively accounting for over 96% of the total plasma density. The number density of polyatomic molecules at such high temperatures was minimal and often not visible in the figure, including species like O_2_, C_2_, O_2_^+^, CO_2_, CO^+^ and MgO. This observation supports the hypothesis proposed in this chapter that “only electrons and atomic cations with the highest valence exist in the plasma at high temperatures,” affirming the hypothesis’s accuracy and practicality in the context of the HLMA algorithm. The figure also shows significant changes in particle number density during the initial stages of iteration. As the homotopy process continued, with the increase in homotopy steps, the difference Δ*t* = *t*_k_-*t*_k−1_ progressively decreased, leading to minimal changes in particle number density, demonstrating that a homotopy factor series composed of proportional steps helps maintain homotopy stability. The PV–LMA exhibited a similar trend to HLMA, but due to fewer iterations, turning points were observed in the particle number density curves, particularly for particles with significant density changes such as C^+^ and O^+^. Additionally, the figure indicates that within the temperature range of 25,000 to 30,000 K, the main plasma components remained electrons and atomic cations.

### 4.4. Comparison of HLMA and PV–LMA

From the calculations and analyses presented in the first three subsections, it is evident that when the auxiliary temperature closely matches the solution temperature, the PV–LMA algorithm demonstrates superior computational speed and efficiency compared to the HLMA method. This advantage aligns with the primary purpose of the PV–LMA algorithm proposed in this chapter, which is to facilitate the batch calculation of plasma number density. However, a critical drawback of PV–LMA is that when there is a significant difference between the number density at the auxiliary temperature and the target composition number density at the solution temperature, large changes in number density during the final iterations can occur. This can increase the likelihood of the algorithm falling into local optimality, leading to potential calculation failures. Although the calculations are successfully completed in the examples discussed in Section 4.2 and Section 4.3, the inherent risk of failure remains higher for PV–LMA in such cases.

**Figure 9:**
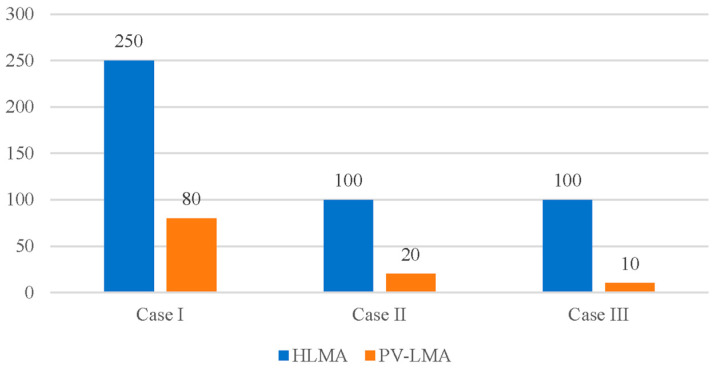
Comparison of the number of calculations required by the HLMA and PV–LMA algorithms.

Figure 9 shows a comparison of the number of calculations required by the HLMA and PV–LMA algorithms in the three examples. Compared with HLMA in the three examples, the computational efficiency of PV–LMA increased by 80%, 90% and 68%, respectively. PV–LMA was more efficient than HLMA. In the batch calculation, the auxiliary temperature was not necessarily high, as long as it was greater than the current solution temperature under the same pressure.

In practical calculations, one effective method for minimizing the gap in number density is to reduce the difference between the solution temperature and the auxiliary temperature. The following guidelines, based on practical experience, may be helpful: for *T* > 15,000 K, set *T*_max_-*T* = 1000 K; for *T* > 8000 K, set *T*_max_-*T* = 500 K; for *T* > 5000 K, set *T*_max_-*T* = 100 K; and for *T* > 3000 K, set *T*_max_-*T* = 50 K. These parameters can effectively shorten the gap between the auxiliary equations and the solution equation, which not only ensures that the solution variable is within a controllable range but also greatly reduces the number of homotopy calculations and improves work efficiency.

## 5. Conclusions

Based on the Saha and Guldberg–Waage equations, an equilibrium composition model for plasma under local thermodynamic and chemical equilibrium conditions was established. This model is formed by combining the stoichiometric number conservation equation, the charge conservation equation, and Dalton’s law of partial pressures, followed by a thorough examination of the model’s singularities. Given that the model represents a nonlinear system of equations with a weakly singular Jacobian matrix and demonstrates sensitivity to initial values in iterative solution algorithms, HLMA is proposed for addressing the equilibrium composition model. To address the limitations of HLMA, such as its extensive computational workload and high iteration count, PV–LMA is introduced. Finally, three computational examples are provided for analysis, yielding the following results.

1. The fundamental concept of “the provision of a reasonable initial value for LMA solution of the *n*-th homotopy equations through (*n-1*)-th homotopy calculation” is particularly well suited for the resolution of weak singular nonlinear equations composed of plasma equilibrium component models. Furthermore, it offers a method for establishing the initial value necessary for the solution of nonlinear equations.
2. Both HLMA and PV–LMA can be used to solve the equilibrium composition model. The calculation accuracy ||***F***|| in all three of the selected examples was less than 1 × 10^−15^.
3. The disparity in the equilibrium coefficients of the Saha and Guldberg–Waage equations is the primary cause of singularity in the nonlinear system of equations representing the plasma equilibrium composition model. A reasonable hypothesis for simplifying this model is that, at high temperatures (e.g., 30,000 K), the plasma predominantly consists of electrons and atomic cations with the highest valence. Under this assumption, accurate solutions can be obtained using the LMA.
4. HLMA is suitable for solving nonlinear systems of equations with singularity when auxiliary equations are available, and the difference *t_k_*-*t_k−1_* between consecutive homotopy control factors is small. This small difference helps maintain the continuity of the HLMA process. In contrast, PV–LMA lacks a control factor sequence with a specialized structure, meaning it cannot ensure the continuity of the calculation process by adjusting Δ*t* = *t_k_*−*t_k−1_* between control factors. This limitation makes PV–LMA more susceptible to calculation failure.
5. In the three examples, HLMA required 100, 100, and 250 calculations, whereas PV–LMA needed only 20, 10, and 80 calculations, respectively. This demonstrates that PV–LMA has a higher computational efficiency compared to HLMA. However, PV–LMA carries an increased risk of falling into local optimality, which can lead to calculation failure in certain cases.

## Figures and Tables

**Table 1:** Comparison between HLMA and PV–LMA.

Method	Auxiliary Equations	Homotopy Sequence	Initial Value	Solution Method
HLMA	High temperature composition model	Geometric series	High temperature composition	LMA
PV–LMA	One-dimensional array	Arithmetic sequence	High temperature composition	LMA

**Table 2:** Particle composition of nitrogen plasma.

Neutral Particles	N_2_, N
Ions	N_2_^+^, N^+^, N^2+^, N^3+^
Electron	e

**Table 3:** Chemical reactions involved in nitrogen plasma.

Serial Number	Chemical Reaction	Serial Number	Chemical Reaction
1	N_2_ ⇌ N + N	2	N_2_ ⇌ N_2_^+^ + e
3	N ⇌ N^+^ + e	4	N^+^ ⇌ N^2+^ + e
5	N^2+^ ⇌ N^3+^ + e		

**Table 4:** Solutions of the auxiliary and the solving equations for N_2_ plasma.

t	e	N	N^+^	N^2+^	N^3+^	N_2_	N_2_^+^
0	8.67 × 10^16^	4.42 × 10^17^	8.30 × 10^16^	1.83 × 10^15^	7.69 × 10^11^	9.56 × 10^14^	8.02 × 10^12^
1	2.47 × 10^16^	6.67 × 10^17^	2.46 × 10^16^	8.31 × 10^13^	3.08 × 10^9^	1.71 × 10^16^	1.99 × 10^13^

**Table 5:** Particle composition of air plasma.

Neutral Particles	N_2_, O_2_, N, O, NO
Ions	N_2_^+^, O_2_^+^, NO^+^, N^+^, N^2+^, N^3+^, O^+^, O^2+^, O^3+^
Electron	e

**Table 6:** Chemical reactions involved in air plasma.

Serial Number	Chemical Reaction	Serial Number	Chemical Reaction
1	N_2_ ⇌ N + N	7	N ⇌ N^+^ + e
2	N_2_ ⇌ N_2_^+^ + e	8	N^+^ ⇌ N^2+^ + e
3	O_2_ ⇌ O + O	9	N^2+^ ⇌ N^3+^ + e
4	O_2_ ⇌ O_2_^+^ + e	10	O ⇌ O^+^ + e
5	NO ⇌ N + O	11	O^+^ ⇌ O^2+^ + e
6	NO ⇌ NO^+^ + e	12	O^2+^ ⇌ O^3+^ + e

**Table 7:** Solutions of auxiliary and solving equations for air plasma.

*t*	e	N	N^+^	N^2+^	N_2_	N_2_^+^
0	6.78 × 10^13^	2.68 × 10^17^	2.31 × 10^15^	4.63 × 10^14^	4.65 × 10^17^	2.01 × 10^15^
1	2.18 × 10^14^	9.00 × 10^16^	3.94 × 10^12^	6.76 × 10^6^	7.24 × 10^17^	2.88 × 10^11^
NO	NO^+^	O	O^+^	O^2+^	O_2_	O_2_^+^
3.31 × 10^14^	1.71 × 10^15^	3.19 × 10^17^	8.74 × 10^14^	5.69 × 10^06^	6.59 × 10^12^	8.8 × 10^13^
8.32 × 10^14^	2.06 × 10^14^	4.08 × 10^17^	7.83 × 10^12^	8.74 × 10^03^	4.92 × 10^13^	2.27 × 10^10^

**Table 8:** Particle composition of Mg−CO plasma.

Neutral Particles	CO, O_2_, C_2_, O, C, Mg, MgO, CO_2_
Ions	O_2_^+^, CO^+^, O^+^, O^2+^, O^3+^, C^+^, C^2+^, C^3+^, Mg^+^, Mg^2+^
Electron	e

**Table 9:** Chemical reactions involved in Mg−CO plasma.

Serial Number	Chemical Reaction	Serial Number	Chemical Reaction
1	CO_2_ ⇌ CO + O	9	C^+^ ⇌ C^2+^ + e
2	CO ⇌ C + O	10	C^2+^ ⇌ C^3+^ + e
3	MgO ⇌ Mg + O	11	O ⇌ O^+^ + e
4	C_2_ ⇌ C + C	12	O^+^ ⇌ O^2+^ + e
5	O_2_ ⇌ O + O	13	O^2+^ ⇌ O^3+^ + e
6	CO ⇌ CO^+^ + e	14	Mg ⇌ Mg^+^ + e
7	O_2_ ⇌ O_2_^+^ + e	15	Mg^+^ ⇌ Mg^2+^ + e
8	C ⇌ C^+^ + e		

**Table 10:** Solutions of auxiliary and solving equations for Mg50%-CO%50 mixture plasma.

*t*	C	C^+^	C^2+^	C^3+^	C_2_	CO
0	2.08 × 10^10^	6.39 × 10^12^	1.15 × 10^15^	2.16 × 10^16^	5.41 × 10^−36^	1.27 × 10^−35^
1	1.32629 × 10^12^	1.8 × 10^14^	7.73 × 10^15^	2 × 10^16^	1.35 × 10^−3^	5.14 × 10^0^
CO^+^	CO_2_	e	Mg	Mg^+^	Mg^2+^	MgO
6.06 × 10^−28^	2.38 × 10^−44^	1.81 × 10^17^	1.6 × 10^10^	1.21 × 10^13^	2.31 × 10^16^	1.25 × 10^−32^
7.98 × 10^02^	3.49 × 10^−10^	2.1 × 10^17^	7.68 × 10^10^	2.72 × 10^13^	2.79 × 10^16^	8.40 × 10^−8^
O	O^+^	O^2+^	O^3+^	O_2_	O_2_^+^	precision
5.07 × 10^10^	1.39645 × 10^13^	6.46 × 10^14^	2.21 × 10^16^	7.59 × 10^−35^	1.22 × 10^−26^	2.25 × 10^−16^
7.32 × 10^12^	6.00223 × 10^14^	4.2 × 10^15^	2.31 × 10^16^	1.27 × 10^1^	7.30 × 10^4^	1.11 × 10^−16^

## Data Availability

The data presented in this study are available on request from the corresponding author.
